# Down-regulation of PKCζ in renal cell carcinoma and its clinicopathological implications

**DOI:** 10.1186/1423-0127-19-39

**Published:** 2012-04-05

**Authors:** Yeong-Shiau Pu, Chao-Yuan Huang, Jyue-Yu Chen, Wang-Yi Kang, Ying-Chu Lin, Yu-Shiang Shiu, Shu-Ju Chuang, Hong-Jeng Yu, Ming-Kuen Lai, Yu-Chieh Tsai, Wen-Jeng Wu, Tzyh-Chyuan Hour

**Affiliations:** 1 Department of Urology, National Taiwan University College of Medicine, Taipei, Taiwan; 2 Department of Oncology, National Taiwan University College of Medicine, Taipei, Taiwan; 3 Institute of Biochemistry, Kaohsiung Medical University, Kaohsiung, Taiwan; 4 Department of Pathology, Kuo General Hospital, Tainan, Taiwan; 5 Faculty of Dentistry, Kaohsiung Medical University, Kaohsiung, Taiwan; 6 Department of Urology, Kaohsiung Medical University Chung-Ho Memorial Hospital, Kaohsiung Medical University, Kaohsiung, Taiwan

**Keywords:** Renal cell carcinoma, PKCζ, Immunohistochemistry, Chemoresistance, Cytotoxicity

## Abstract

**Background:**

Metastatic renal cell carcinoma (RCC) is highly resistant to systemic chemotherapy. Unfortunately, nearly all patients die of the metastatic and chemoresistant RCC. Recent studies have shown the atypical PKCζ is an important regulator of tumorigenesis. However, the correlation between PKC**ζ **expression and the clinical outcome in RCC patients is unclear. We examined the level of PKCζ expression in human RCC.

**Methods:**

PKCζ mRNA and protein expressions were examined by real-time polymerase chain reaction (PCR) and immunohistochemistry (IHC) respectively in RCC tissues of 144 patients. Cellular cytotoxicity and proliferation were assessed by MTT.

**Results:**

PKCζ expression was significantly higher in normal than in cancerous tissues (*P *< 0.0001) by real-time PCR and IHC. Similarly, PKCζ expression was down-regulated in four renal cancer cell lines compared to immortalized benign renal tubular cells. Interestingly, an increase of PKCζ expression was associated with the elevated tumor grade (*P *= 0.04), but no such association was found in TNM stage (*P *= 0.13). Tumors with higher PKCζ expression were associated with tumor size (*P *= 0.048). Expression of higher PKCζ found a poor survival in patients with high tumor grade. Down-regulation of PKCζ showed the significant chemoresistance in RCC cell lines. Inactivation of PKCζ expression enhanced cellular resistance to cisplatin and paclitaxel, and proliferation in HK-2 cells by specific PKC**ζ **siRNA and inhibitor.

**Conclusions:**

PKCζ expression was associated with tumorigenesis and chemoresistance in RCC.

## Background

The incidence of renal cell carcinoma (RCC) is increasing worldwide [1]. RCC mainly arises from renal tubular epithelia [2]. Surgical resection of the diseased tissue has been considered the only curative treatment [3]. Metastatic RCC (mRCC) is generally resistant to chemotherapy and hormonal therapy and marginally sensitive to immunotherapy [4]. Although several promising therapeutic strategies are now available for treating patients with mRCC, nearly all patients die of the metastatic disease. Research is ongoing to identify RCC-specific biomarkers that can improve early diagnosis, surveillance of tumor progression, and prediction of patient prognosis [5]. Markers such as growth factors, laminin, p53 mutations, and others, have been recently examined [6-8]. Unfortunately, none of these markers appear superior to the traditional staging and grading systems. RCC is also characterized by a high resistance to tumor cell apoptosis both intrinsic and induced by radiation or systemic therapies, including chemotherapy and immunotherapy [9-13]. However, the mechanisms of this resistance have not been elucidated.

The PKC family includes at least 11 isoforms of closely related serine/threonine protein kinases that regulate important cellular processes, including proliferation, differentiation, and apoptosis [14,15]. Members of this family are classified into three subfamilies according to their sequence homology and activating cofactor requirements [14]. The conventional PKCs (α, βI, βII, and γ) are activated by calcium (Ca^+2^) and 1,2-diacyl-*sn*-glycerol, whereas members of the novel class of PKC (δ, ε, θ, and η) are activated by 1,2-diacyl-*sn*-glycerol, but are Ca^+2 ^independent. The atypical PKC isoforms (λ, ζ and ι) are both Ca^+2^- and 1,2-diacyl-*s*n-glycerol independent [15]. Although PKC isoforms have overlapping substrate specificities *in vitro *[16], these kinases display distinct patterns of tissue expression and intracellular localizations that likely reflect unique isoform specific functions [17,18].

PKC has been associated with tumor promotion, progression, invasion, and metastasis [16,19]. Accumulated results have demonstrated PKCζ plays an important role in human tumorigenesis of the colon, skin, prostate, lung, and bladder [19-23]. Mustafi et al. suggested PKCζ might inhibit cancer cell growth and enhance differentiation and apoptosis [19]. Similarly, the down-regulation of PKCζ may contribute to skin tumorigenesis by releasing constraints on Akt/PKB activity, proceeding during skin tumor promotion and progression [24]. Inoue et al. also suggested the PKCζ-mediated mammalian target of the rapamycin/S6 kinase pathway plays an important role in the transition of androgen-dependent to androgen-independent prostate cancer cells [21]. In lung cancer, PKCζ plays an important role in Par4 inhibition Akt activity and Ras-induced tumorigenesis. Additionally, PKCζ was involved in suppressing melanoma cell migration and Rho-dependent prostate cancer cell proliferation and apoptosis [24,25]. In aggregate, these data support the hypothesis PKCζ may act as a novel tumor suppressor in tumorigenesis. Conversely, recent studies have indicated PKCζ played an oncogenic role to promote tumorgenesis and inhibit apoptosis in breast cancer, head and neck cancer [26,27].

The expression profile of PKCζ in the progression of renal tumorigenesis has not been previously studied. To address this question, we explored the role of PKCζ expression in human RCC.

## Methods

### Chemicals and reagents

Anti-PKCζ and anti-α-tubulin antibodies were purchased from Santa Cruz Biotechnology (Santa Cruz, CA) and Oncogene Science (Cambridge, MA), respectively. Protein kinase Cζ pseudosubstrate inhibitor was obtained from BioSource International Inc (Camarillo, CA). Specific PKCζ siRNA was synthesized from Invitrogen (Invitrogen, Carlsbad, CA). Cisplatin and paclitaxel were purchased from Sigma Chemical Co. (St. Louis, MO, USA).

### Cell culture

HK-2 is an immortalized cell line derived from human proximal renal tubular cells. HK-2 cells were cultured in a keratinocyte-serum free medium containing 5 ng/ml recombinant epidermal growth factor and 40 μg/ml bovine pituitary extract. The four utilized RCC cell lines were 769-P, 786-O and ACHN cells, maintained in RPMI-1640 medium, A498 cells were maintained in MEM medium. These medium were supplemented with 10% fetal calf serum (FCS) (Invitrogen), 100 μg/ml penicillin-streptomycin, and 1% glutamine.

### Cytotoxic assay

Cellular chemosensitivity to cisplatin and paclitaxel were determined using a modified 3-(4,5-dimethylthiazo-2-yl)-2,5-diphenyl tetrazolium (MTT, Sigma Chemical Co., ST. Louis, MO) assay *in vitro *[28]. In brief, cells were treated with anti-cancer drugs (each in 100 μl of culture medium) simultaneously and incubated for 72 h. At 72 h, 50 μl of MTT (2 mg/ml) was added to each well and incubated for 2.5 h. Blue formazan crystals thus formed were pelleted to the bottom of the well by centrifugation, separated from the supernatant and dissolved in 150 μl of dimethylsulfoxymide. The optical density at 492 nm was determined by absorbance spectrometry using a microplate reader (MRX-2, Dynex Technologies, Inc., Chantilly, VA). Three separate experiments with triplicate data were performed to obtain mean cell viability.

### PKCζ knock-down assay

The specific Stealth PKCζ small interfering RNA (siRNA) was synthesized from Invitrogen for PKCζ knock-down. PKCζ target siRNA sequence was 5'-GACAUGAACACAGAGGACUACCUUUU-3'. HK-2 cells (2 × 10^5^) were plated into 10-cm plate overnight. Then cells were transfected with 100 nM of PKCζ siRNA with lipofectamine (Invitrogen) reagent for 24 h. Chemosensitivity of HK-2 cells to cisplatin and paclitaxel was examined using MTT assay after siRNA transfection.

### Clinicopathological characteristics

This study had 144 patients with RCC, 98 males and 46 females with a mean age of 62.2 ± 12.2 years. Each pair of tissues included a RCC tumor portion and normal-looking renal cortical tissue from the same patient. These specimens were obtained from nephrectomies carried out at the National Taiwan University Hospital (NTUH). Fuhrman's nuclear grading system from I to IV was used [29]. Tumors were staged according to the TNM system and histologically classified according to WHO guidelines [30]. The clinicopathological characteristics of the tumors are summarized in Table 1. Approval from the Institutional Review Boards of NTUH and Kaohsiung Medical University were obtained and informed consent was received from all participating patients.

**Table 1:** Immunostaining expression of PKCζ in normal parenchymal and RCC tissues

	Patients	PKCζ protein expression (mean score ± SE)		
		
Characteristic	No. (%)	Normal renal tubular cells	RCC	*P *value*
Total	144 (100)	161.1 ± 4.5	103.6 ± 4.5	< 0.0001
Sex				
Male	98 (68)	158.5 ± 5.4	103.3 ± 5.5	< 0.0001
Female	46 (32)	166.5 ± 8.0	104.3 ± 7.9	< 0.0001
*P *value^†^		0.41	0.91	
Grade				
I	19 (13)	166.8 ± 13.1	79.5 ± 10.6	< 0.0001
II	64 (45)	160.8 ± 6.7	101.7 ± 6.8	< 0.0001
III	32 (22)	168.1 ± 8.9	113.8 ± 9.1	< 0.0001
IV	15 (10.3)	166 ± 10.9	111.3 ± 15.1	0.009
ND	14 (9.7)	132.9 ± 18.1	109.3 ± 17.2	0.3
*P *for ANOVA^‡^		0.91	0.14	
*P *for trend^¶^		0.79	0.04	
Stage				
Organ-confined (T1-2N0 M0)	95 (66)	159.1 *± *5.8	102.1 *± *5.6	< 0.0001
Locally advanced (T3-4 N0 M0)	31 (22)	160 *± *10.2	103.9 ± 8.5	< 0.0001
Metastatic (N1-2 or M1)	17 (11.3)	162.4 ± 11.3	115.3 ± 15.7	0.032
ND	1 (0.7)	200	50	
*P *for ANOVA^‡^		0.97	0.67	
*P *for trend^¶^		0.41	0.13	
Histological type				
Conventional	108 (75)	163.1 ± 4.9	98.1 ± 4.7	< 0.0001
Non-conventional	36 (25)	152.2 ± 11.2	115.6 ± 11.5	0.0082
*P *value^†^		0.31	0.098	

NOTE: PKCζ protein expression was examined by immunohistochemistry on a scale from 0 to 200 (percent positive cells × staining intensity). Non-conventional type include papillary, chromophobe, sarcomatoid and collecting duct. Abbreviations: RCC, renal cell carcinoma; SE, standard error; TNM, tumor-node-metastasis; ND, not determined. *: paired sample t test, †: Independent-sample t test for two groups, ‡: One-way ANOVA for three or more groups, ¶: Test for linear trend based on the continuous variable

### Real-time PCR

Primers were synthesized to encompass a specific segment of the cDNA sequence of the PKCζ (forward primer, 5'-AGAAAGAGCTGGTGCATGATGAC-3'; reverse primer, 5'-TGCTGGATGCCTGCTCAA-3'), and GADPH (forward primer, 5'-TCTCCTCTGACTTCAACAGCGAC-3'; reverse primer, 5'-CCCTGTTGCTGTAGCCAAATTC-3')(Invitrogen). A master-mix of the following reaction components was prepared with the indicated final concentrations: 6.4 μl of water, 1.2 μl of MgCl_2 _(4 mM), 1 μl of forward primer (0.4 μM), 1 μl of reverse primer (0.4 μM) and 12.5 μl of LightCycler Fast Start DNA Master SYBR Green I (PE Applied Biosystems, Foster City, CA, USA). Nine microliters of the master-mix was added to each well of a 96-well plate and 1 μl containing 50 ng cDNA, was added as the PCR template. The corresponding cDNA fragments were denatured at 95°C for 15 sec and annealed at 60°C for 1 min. At the completion of 40-cycle, melting curve analysis was performed to establish the specificity of the amplicons produced. A ratio of specific mRNA/GADPH (GADPH as a respective control) amplification was then calculated (ΔC_t _vaule = C_tPKCζ _- C_tGADPH_), to correct for any differences in efficiency. The fold changes were calculated with the ΔΔC_t _method (the total ΔΔC_t _= fold of cancerous/normal tissue gene level), using normal tissue (for tissue samples) or HK-2 (for cell lines) as the control.

### Immunohistochemistry (IHC)

Immunostaining was performed as described previously [19]. In brief, the sections were de-paraffinized in xylene and rehydrated through graded alcohols, then boiled in 0.01 M citrate buffer (pH 6.0) for 10 min. Hydrogen peroxide, 0.3%, was added to block any endogenous peroxidase activity. The sections were incubated with anti-PKCζ antibody used at a 1:600 dilution at 4°C overnight. Horseradish peroxidase (HRP) polymer conjugated was used as a second antibody to avoid contaminating the endogenous biotin or streptavidin (Zymed). After washing, the antigen-antibody complex was applied and stained with diaminobenzidine (Golden Bridge, Mukilteo, WA). Counterstaining was performed lightly with hematoxylin. The expression of PKCζ was evaluated according to the ratio of positive cells per specimen and the staining intensity, as described previously [31]. A semiquantitative procedure was used to generate an IHC score for each tissue section. The ratio of positive cells per specimen was evaluated quantitatively and scored from 0 ~ 100% of the cells examined. Intensity was graded as follows: 0, no signal; 1, weak; and 2, strong staining. A total score of 0 to 200 was finally calculated as the percentage of positive cells × staining intensity. The evaluation of immunostaining was performed by a single pathologist (W. Y. K), who was unaware of the fate of the patient or the tissue site.

### Western blotting analysis

Cell extracts (50 μg) were separated on 10% SDS-polyacrylamide gels and transferred to immobilon polyvinylidene difluoride membranes (Millipore, Bedford, MA). After blocking, the membranes were incubated with human specific anti-PKCζ (Santa Cruz Biotechnology) polyclonal antibody at 4°C for 12 h, followed by the horseradish peroxidase-labeled second antibody, and developed with the ECL system (Santa Cruz Biotechnology).

### cDNA microarray

Total RNAs were extracted from 3 pairs of RCC and adjacent normal kidney tissues from 3 patients. Thirty micrograms RNA were reversely transcribed to cDNA by SuperScript^® ^II reverse transcriptase in the presence of aminoallyl-dUTPs (Invitrogen). The cDNA was labeled by either Cy3 or Cy5 at the aminoallyl-dUTP sites using a coupling reaction. Cye dye coupling efficiency was determined by UV spectrophotometer to determine the absorbance at 260 nm for DNA, 550 nm for Cy3 and 650 nm for Cy5. Labeled cDNA was hybridized to the oligonucleotide probes of a TMSEC microarray (Taiwan Genome Sciences, Taipei, Taiwan) at 50°C for overnight. The microarray was washed, and the emission signals were scanned using the GenePix 4000A Fluorescent Scanner (Axon Instruments, South San Francisco, CA). Each time point and condition was repeated typically on at least three to five occasions. Microarrays were analyzed with the Scanalyze program, as written by Taiwan Genome Sciences Inc, to determine the fluorescent intensities of the Cy5 and Cy3 for each spot. The fluorescence intensities were normalized by applying a scaling factor so that the median fluorescence ratio of all spots with detectable signals above background on each microarray is 1.0. The data are then filtered so that only spots with intensities that are three times greater than background in either channel are included in the analysis. Only those spots that displayed a 2-fold or greater difference in fluorescence intensities between the two dyes are used to generate gene clusters. A list of ~3,000 genes with altered expression (*P *< 0.001) was generated using these analyses criteria.

### Statistical analysis

The levels of PKCζ mRNA between the normal and RCC tissues were compared by the paired t-test. Data are presented as the mean ± standard error of the means (SEM). Independent-sample t test, One-way ANOVA and linear trend test were used to compare protein expression determined by IHC analysis. Kaplan-Meier method was used to estimate the probability of overall survival. The log-rank test was performed to examine the association of PKCζ with overall survival. All tests were two-sided with *P *< 0.05 being statistically significant.

## Results

### Down-regulation of PKCζ in renal cell carcinoma (RCC)

**Figure 1:**
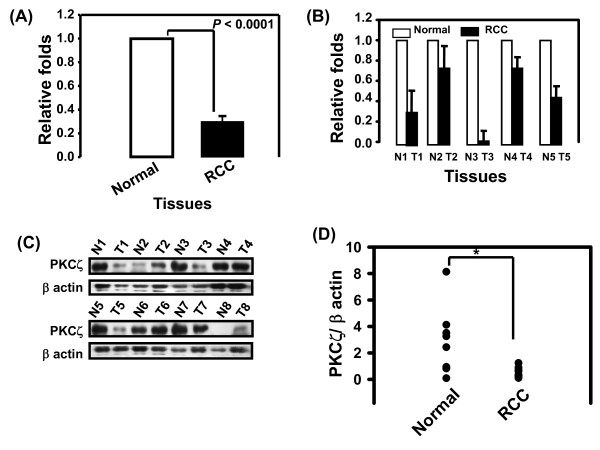
**Down-regulation of PKCζ in RCC tissues**. **(A) **Expression of PKCζ mRNA in 3 pairs of RCC and adjacent normal kidney tissues from 3 patients by cDNA microarray analysis. Fold change compared with normal kidney tissues. Paired sample t test was used to compare PKCζ expression in the normal and RCC tissues (*P < 0.001). **(B) **Real-time PCR analysis of PKCζ in 5 pairs of RCC (T) and adjacent normal kidney tissues (N) from 5 patients. **(C) **Expression of PKCζ protein in 8 pairs of normal and RCC tissues by Western blotting analysis. β-actin was used as an internal control **(D) **The ration of protein intensity of PKC ζ/β-actin from Figure 1(C) was examined by densitometer analysis. Paired sample t test was used to compare PKCζ expression in the normal and RCC tissues (*P < 0.05).

Three pairs of RCC and normal renal parenchymal tissues from the same patients were analyzed using cDNA microarray, showing PKCζ gene expression in the cancerous tissues was 3-fold lower than in the normal tissues (*P *< 0.0001, Figure 1A). PKCζ mRNA expression was determined with real-time PCR using five pairs of tissues including the three used in the cDNA microarray experiment (Figure 1B). PKCζ mRNA levels were significantly higher in normal than in cancerous tissues (Figure 1B). Similarly, the expression of PKCζ protein was estimated in eight pairs of tissues by Western blotting (Figure 1C and 1D).

### Immunohistochemical analysis of PKCζ expression in paired tissues

**Figure 2:**
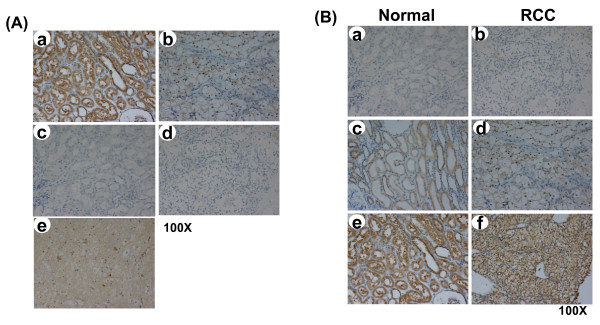
**Down-regulation of PKCζ in human conventional RCC tissues by Immunohistochemical staining**. **(A) **Tissue sections of normal parenchymal tissues (a, c) or RCC (b, d) were from the same patient. The polyclonal anti-PKCζ antibody was used to stain paraffin sections (a, b). The negative control omitted the primary antibody (c, d). The brain tissue was used as a positive control for PKCζ expression (e). The tumor grade and stage in (b) and (d): grade I and TNM stage T_2_N_0_M_0_. Original magnification, × 100. **(B) **Representative immunohistochemical staining of PKCζ expression in normal parenchymal and RCC tissues. (a) and (b), negative staining (0+); (c) and (d), weakly positive staining (1+); (e) and (f), strongly positive staining (2+), The tumor grade and stage in (b): grade I and TNM stage T_1_N_0_M_0_, in (d): grade II and TNM stage T_1b_N_0_M_0 _and (f): grade IV and TNM stage T_3a_N_1_M_1 _(100 **× **magnification).

The expression of PKCζ protein was estimated in 144 pairs of tissues by immunohistochemistry (IHC). Specific staining of PKCζ was observed in the cytoplasm. The PKCζ protein staining was strongly positive in normal renal tubular cells (Figure 2A-a) but weakly positive in most cancerous epithelia (Figure 2A-b). IHC expression levels were further quantified on a scale from 0 to 200 (Figure 2B and Table 1). The normal renal tissues had markedly elevated scores of 161.1 ± 4.5 compared to the RCC tissues scores of 103.6 ± 4.5 (*P *< 0.001), as presented in Table 1. Interestingly, it showed an increase of PKCζ protein expression was associated with elevated tumor grade (*P *= 0.04), but no such association was found in TNM stage (*P *= 0.13) using linear trend test (Table 1). However, as shown in Table 1, PKCζ protein levels in the RCC tissues did not differ between genders, tumor grades, TNM stages, or histological types (all *Ps *> 0.05). Based on tumor size from AJCC TNM classification for renal cell carcinoma, we found RCC patients with bigger tumor size had higher intensity of PKCζ expression (*P *= 0.048) (Table 2). Although there was no significant correlation between PKCζ expression and invasive growth characteristic (*P *= 0.67), but it showed an increased trend for PKCζ expression in RCC (Table 1).

**Table 2:** Intensity of PKCζ expression was associated with tumor size in RCC

Characteristics	Intensity of PKCζ (N%)^a^				
	
Tumor size	0	1	2	χ^2^*	*P *value
≤4 cm	0	42	17	9.57	0.048
	(0)	(48.8)	(34.7)		
4-7 cm	2	25	13		
	(100)	(29.1)	(26.5)		
> 7 cm	0	19	19		
	(0)	(22.1)	(38.8)		

a: patient numbers% (N%)

*: the association of PKCζ intensity with tumor size was analyzed by Chi-Square test

### The prognostic significance of PKCζ expression in RCC

**Figure 3:**
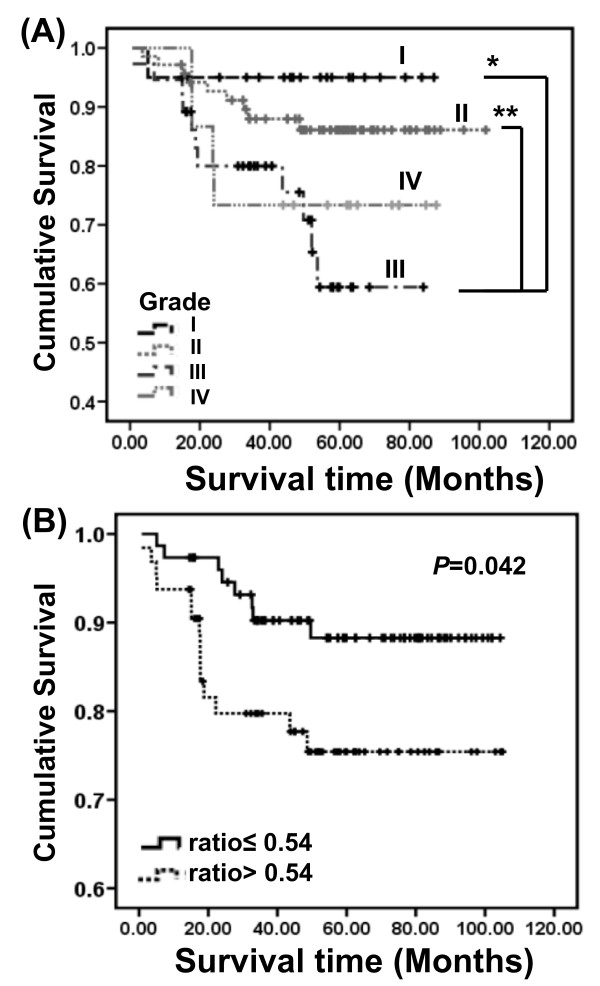
**The prognostic survival of PKCζ in RCC**. **(A) **High tumor grade was associated with short survival time in RCC patients. Tumor grade III was significantly associated with poorer outcome than grade I (*P *= 0.032) and II (*P *= 0.016) on 5-year survival, respectively. **(B) **High expression of PKCζ was associated with poorer clinical prognosis. The ratio of PKCζ expression (cancer/normal ratio) was PKCζ score of cancer compared with normal tissue from the same patient. The median value of all RCC patients was 0.54. PKCζ ratio > 0.54 had significantly poor survival than ratio ≤ 0.54 on 5-year survival in RCC (*P *= 0.042).

We evaluate the relationship between tumor grade and 5-year survival in a population of patients with RCC. As shown in Figure 3A, the tumor grade III was significantly associated with poor outcome than grade I (*P *= 0.032) and II (*P *= 0.016) on 5-year survival, respectively. Thus, higher expression of PKCζ was associated with short survival time in RCC patients with higher tumor grade. However, grade IV showed no significant 5-year survival between grade I and grade II. The ratio of PKCζ expression (cancer/normal ratio) was PKCζ score of cancer compared with normal tissue from the same patient. The median value of all RCC patients was 0.54. Interestingly, we found the PKCζ ratio > 0.54 had significantly poor survival than ratio ≤ 0.54 on 5-year survival in RCC (*P *= 0.042) (Figure 3B).

### Down-regulation of PKCζ showed the higher chemoresistance in RCC cell lines

**Figure 4:**
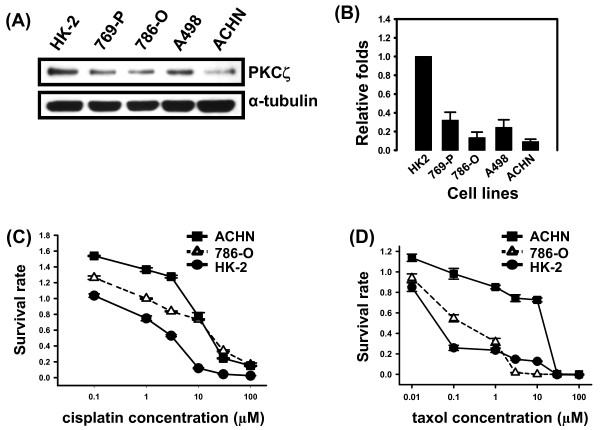
**Down-regulation of PKCζ showed the higher chemoresistance in RCC cell lines**. **(A) **PKCζ protein expression in HK-2 and 4 RCC cell lines by Western blotting. α-tubulin was used as an internal control. **(B) **The intensity of PKCζ protein in figure 4(A) was examined by densitometer analysis. Fold changes were normalized against the expression of PKCζ protein in HK-2 cells. Chemosensivity of HK-2, 786-O and ACHN cells to **(C) **cisplatin and **(D) **paclitaxel were examined by MTT assay, respectively.

PKCζ protein levels were significantly reduced in the four RCC cell lines compared to HK-2 the benign immortalized cell line by Western blotting (Figure 4A and 4B), respectively. Similarly, PKCζ gene levels were decreased in these RCC cell lines as estimated (data not shown). We identified the molecular role of PKCζ in RCC cells. The higher expression of PKCζ in HK-2 was with higher sensitivity to cisplatin and paclitaxel than in the 786-O and ACHN cell lines, respectively (Figure 4C and 4D).

### Inactivation of PKCζ expression enhanced cellular chemoresistance and proliferation

**Figure 5:**
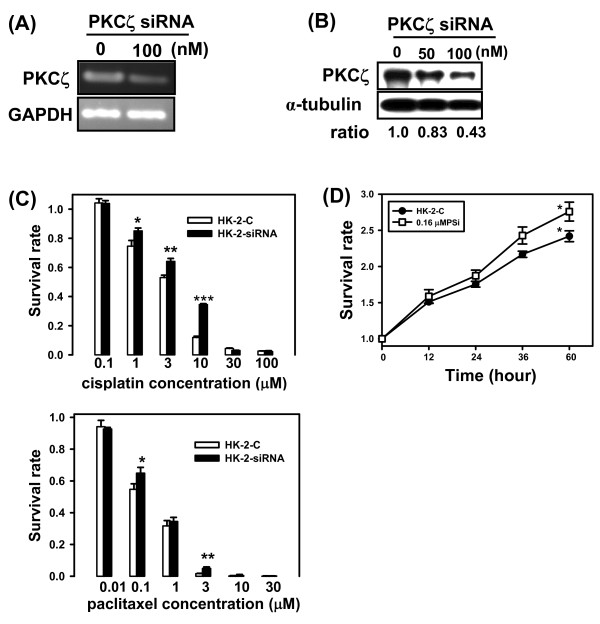
**Inhibition of PKCζ expression led to increased chemoresistance and cell proliferation in HK-2 cells**. **(A) **siRNA-mediated suppression of PKCζ gene expression in HK-2 cells. PKCζ siRNA (100 nM) were transfected into parental HK-2 cells. Note, the level of targeted PKCζ gene was significantly reduced by > 50% using RT-PCR analysis. **(B) **Similarly, the level of PKCζ protein was significantly inhibited by 57% at 100 nm siRNA using Western blotting analysis. C: control group, lipofectamine only, and α-tubulin was used as an internal control. **(C) **Parental HK-2 cells were treated 100 nm PKCζ siRNA (HK-2-siRNA) or lipofectamine only (HK-2-C), respectively for 24 hours. Chemosensivity of HK-2-C (IC_50 _= 2.05 μM) and HK-2-siRNA (IC_50 _= 4.02 μM) cells to cisplatin were examined by MTT assay. The IC_50 _value was a near 2-fold increase in HK-2-siRNA compared to HK-2-C. Similar results, chemosensivity of HK-2-C (IC_50 _= 0.09 μM) and HK-2-siRNA (IC_50 _= 0.17 μM) cells to paclitaxel were examined by MTT assay. The IC_50 _value was a near 2-fold increase in HK-2-siRNA compared to HK-2-C. P* < 0.05, P** < 0.005 and P*** < 0.001. **(D) **Parental HK-2 cells were treated with 0.16 μM PKCζ pseudosubstrate. After 60 h, cell proliferation was assessed by MTT assay. Points, means of two experiments plated in replicates of six; Data are presented as the mean ± standard error of the means (SEM). P* < 0.05, compared with untreated cells.

Knockdown of PKCζ expression in HK-2 by specific PKCζ siRNA (Figure 5A and 5B), indicated down-regulated PKCζ expression enhanced HK-2 cells the resistance to cisplatin at the concentrations of 1, 3 and 10 μM, respectively (P* < 0.05, P** < 0.005 and P*** < 0.001) (Figure 5C). Chemosensivity of HK-2-C (IC_50 _= 2.05 μM) and HK-2-siRNA (IC_50 _= 4.02 μM) cells to cisplatin were examined by MTT assay. The IC_50 _value was a near 2-fold increase in HK-2-siRNA compared to HK-2-C. Similar results, chemosensivity of HK-2-C (IC_50 _= 0.09 μM) and HK-2-siRNA (IC_50 _= 0.17 μM) cells to paclitaxel were examined by MTT assay (Figure 5C). The IC_50 _value was a near 2-fold increase in HK-2-siRNA compared to HK-2-C. Herein, knockdown of PKCζ expression enhanced HK-2 cells the resistance to paclitaxel at the concentrations of 0.1 and 3 μM, respectively (P* < 0.05 and P** < 0.005). Cell permeable protein kinase Cζ pseudosubstrate inhibitor was used to inhibit the PKCζ activity of HK-2 cells. Interestingly, we also found inactivation of PKCζ by specific inhibitor of 0.16 μM PKCζ pseudosubstrate could promote significantly cell growth in HK-2 cells (*P *< 0.05) (Figure 5D).

## Discussion

PKCζ mRNA levels were down-regulated in human renal cancerous tissues compared with their normal tissues, as assessed by cDNA microarray analysis. Additional experimental results confirmed both PKCζ protein and mRNA levels were reduced in RCC tissues and cell lines, suggesting PKCζ may serve as a biomarker for renal tumorigenesis. Interestingly, we found an increase of PKCζ protein expression was associated with elevated tumor grade, but no such association was found in TNM stage using linear trend test. However, since the PKCζ expression level did not differ between the varied genders, tumor grades, TNM stages or histological types of RCC. We also found PKCζ was involved in cell growth and chemoresistance of RCC in this study. We concluded PKCζ played an important role in the tuomrigenesis and chemoresistance of RCC. The phenomena might indicate expression of PKCζ down-regulation could promote at early stage of RCC progression. Therefore, it was not surprising that expression of PKCζ down-regulation in most cancerous compared with normal at the same section.

Recent studies have suggested PKCζ might inhibit cancer cell growth, and enhance differentiation and apoptosis [19,32]. Loss of this growth regulation is a characteristic feature of malignant transformation [33]. Interestingly, Wali et al. previously demonstrated that PKCζ was downregulated in azoxymethane-induced colonic carcinogenesis [32]. They also found three structurally unrelated agents inhibited azoxymethane tumorigenesis and concomitantly prevented PKC-ζ down-regulation in these tumors. Recently, Mustafi et al. have similar finding that PKCζ was up-regulated expression during normal colonocyte maturation, and loss of expression in colonic tumorigenesis, as well as PKCζ preservation by chemopreventive agents. Thus they suggest that PKCζ may inhibit cancer cell growth and enhance differentiation and apoptosis [19]. We have searched the public domain from of NCBI GEO profiles for analysis of the PKCζ RNA level in human RCC microarray. Similarly, there two data profiles were with the same as our experimental conditions in our study demonstrated PKCζ expression was significantly lower in RCC tissues than that in normal tissues. (GDS2880/202178_at/PRKCZ/Homo sapiens and GDS505/202178_at/PRKCZ/Homo sapiens). Herein, the present study showed that PKCζ expression was significantly down-regulation in RCC tissues and cell lines, and inactivation of PKCζ could promote the cell growth in HK-2 cell line. Based on these findings, we speculated that PKCζ mediated interactions between integrins and extracellular matrix that participated in cell-cell and cell-basement membrane signaling. Because PKCζ played a critical role in tight junction biogenesis in other cells [33], down-regulation of this atypical isoform in renal tumorigenesis might abrogate normal growth inhibition mediated by cell-cell contact. Therefore, down-regulation of PKCζ in renal tumorigenesis might abrogate normal growth inhibition mediated by cell-cell contact. These results indicate PKCζ may be associated with renal tubular tumorgenesis. In aggregate, these data support the hypothesis PKCζ may act as a novel tumor suppressor in renal tumorigenesis. However, some studies have shown PKCζ up-regulation and activation in tumorigenesis [34-36], conflicting with the results in this study, but not all reports have thoroughly confirmed this observation in human RCC [37]. Herein, our results revealed down-regulation of PKCζ may be an important phenomenon in the tumor progression from normal to precancer cells or in situ early cancer status. We hypothesized that known-down or inhibition of PKCζ expression in the precancer lesions may therefore abolish the growth suppressive function of this protein and enable rapid proliferation and promoting early cancer development. However, up-regulation of PKCζ expression from a precancer lesion to cancer may imply a differing promotion rather than suppressive function of PKCζ in this stage of disease progression.

An increase of tumor grade was known to be associated with poor survival. Although, the level of PKCζ gene expression was lower in cancer tissue compared with that in paired normal tissue, an increase of PKCζ protein expression in cancer tissues was associated with higher tumor grade using linear trend test (Table 1). However, no such association was found in paired normal tissue samples and those with different TNM cancer stages. These data could partially explain why the high expression of PKCζ was associated with poorer clinical prognosis with ratio (cancer/normal) more 0.54 had significantly poor survival.

We also showed the higher intensity of PKCζ expression in RCC was associated with greater tumor size (Table 2). The tumor size at a given stage was regulated and balanced by specific interactions of multiple factors, for example cellular proliferation, apoptosis and angiogenesis. Previous studies have shown the induction of hypoxic stress, altered microenvironmental pH and growth factors and removed ECM contacts become prominent with the expansion of tumor size [38]. Previously, Datta et al. have demonstrated that PKCζ down-regulated the total mRNA level of FIH-1 and thereby helped HIF-2/HIF-1α to be activated in RCC cell lines [37]. Due to HIF involved in various physiological processes of renal tuomrigenesis during hypoxia, so we predicted PKCζ might play a protective role in the inner of tumor mass from hypoxia-mediated damage in RCC. Whether PKCζ expression played a contributory role or reflected a consequence of increased RCC tumor mass, however still remained to be further studied.

Metastatic RCC is usually with highly resistant to systemic chemotherapy, and nearly all patients die of this metastatic disease [4]. However, the mechanisms of RCC chemoresistance are not well-known studied now. The involvement of PKC pathways in resistance to chemotherapeutic treatments has been studied for quite a long time. The two mechanisms that mainly account for the participation of PKCs in chemotherapeutic resistance are: (a) the modulation of multi-drug transporters, and (b) the regulation of apoptosis [39]. Further studies have revealed PKC activation is not always associated with resistance, but can also increase sensitivity to chemotherapy. For example, while expression of PKCα and PKCγ increases the resistance of human uterine sarcoma cells to paclitaxel, elevated expression of PKCι/λ, leads to reversal of the resistance [40]. Likewise, PKCα overexpression has been associated with increased multi-drug resistance expression. However, in the MDA-MB-231 breast cancer cells, expression of PKCα confers sensitivity to retinoic acid treatment [41]. Interestingly, PKCδ also shows disparate effects in the apoptotic responses to anti-cancer drugs. It seems to act as an anti-apoptotic mediator. Therefore, these findings underlie the relevance of isoform specificity rather than total PKC activity, in the cellular responses to anticancer drugs [42]. Our study also found down-regulation of PKCζ showed the higher chemoresistance in RCC cell lines. PKC**ζ **down-regulation may be associated with chemoresistance in RCC. However, further studies are necessary for elucidating the chemoresistant mechanism of PKC**ζ **of RCC.

## Conclusions

PKC**ζ **expression was shown to be significantly down-regulated in RCC tissues and cell lines, and it may be associated with tumorigenesis and chemoresistance in RCC. To our knowledge, this is the first study showing an association of PKC**ζ **expression levels and renal tubular tumorigenesis. These findings suggest PKC**ζ **may be a potential biomarker for renal tumorigenesis. PKC**ζ **may become the focus of a new strategy of targeted therapy for renal cancer by restoring the PKC**ζ **expression in precancerous or cancerous cells. However, the uncovering mechanisms remained to be further studied.

## Competing interests

The authors declare that they have no competing interests.

## Authors' contributions

YS and CY planed the design of the study, participated in tissue collection and clinicopathological classification. JY performed Immunohistochemical stain and Western blotting. WY participated in the evaluation of immunostaining score. YC and SJ conceived of the study, performed the statistical analysis and assisted to draft the manuscript. YS contributed to the data of PKC**ζ **knock-down by siRNA in HK-2 cells. HJ, MK, YC and WJ participated in tissue collection, clinicopathological classification and assisted to draft the manuscript. TC conducted the experiments, wrote the manuscript, and participated in its design and coordination. All authors read and approved the final manuscript.
